# A qualitative study and preliminary model of living with dementia and incontinence at home: beyond containment

**DOI:** 10.1093/ageing/afab221

**Published:** 2021-11-13

**Authors:** Catherine Murphy, Christine de Laine, Margaret Macaulay, Miriam Avery, Mandy Fader

**Affiliations:** School of Health Sciences, University of Southampton, Southampton, UK; School of Health Sciences, University of Southampton, Southampton, UK; School of Health Sciences, University of Southampton, Southampton, UK; School of Health Sciences, University of Southampton, Southampton, UK; School of Health Sciences, University of Southampton, Southampton, UK

**Keywords:** incontinence, dementia, toilet-use, independent living, family carers, older people, qualitative

## Abstract

**Background:**

most people living with dementia (PLWD) will develop incontinence problems with associated harmful consequences. Well-contained incontinence is often the main treatment goal. It would therefore be expected that poorly contained incontinence would have a negative impact.

**Aim:**

to investigate differences in how well-contained or poorly contained incontinence impacts on the experience of living with incontinence for PLWD at home and their carers.

**Design:**

secondary analysis of a qualitative study.

**Methods:**

semi-structured interviews were undertaken with PLWD, carers and healthcare professionals (continence or dementia nurses). PLWD and carers were recruited via www.joindementiaresearch.nihr.ac.uk and via dementia/carer groups. Nurses were recruited via their employers. Interviews were recorded and transcribed verbatim. Framework analysis was used.

**Results:**

forty-five people (twenty-six carers, two PLWD, nine continence nurses and eight dementia nurses) participated. Despite poorly contained incontinence, some PLWD/carer dyads appeared relatively unaffected by incontinence. Conversely, one or both members of some dyads who achieved good containment found incontinence care highly challenging. Four themes were identified, together forming a preliminary model of incontinence containment and impact, as follows:

1. Well-contained incontinence, lower negative impact
2. Well-contained incontinence, higher negative impact
3. Poorly contained incontinence, higher negative impact
4. Poorly contained incontinence, lower negative impact

**Conclusion:**

reliable containment is an important goal for PLWD living at home and their carers, but it is not the only goal. Other factors, such as behaviours that challenge or carer coping strategies, can mean that even well-contained incontinence can have a negative impact. This paper proposes a preliminary model for evaluation.

## Key Points

- Reliable containment is not the only goal for people living with dementia (PLWD) living at home and their carers.
- Even well-contained incontinence can have a negative impact on people living with dementia (PLWD) and their carers.
- A preliminary model offers a framework to understand how incontinence might influence people living with dementia (PLWD) and carers differently.

## Introduction

Most of the 50 million people living with dementia (PLWD) live in their own home with support provided by family members or friends (‘carers’) [1]. Unfortunately, the majority of PLWD will develop bladder or bowel incontinence or toilet-use problems at some point and are at considerably higher risk of these problems than someone of the same age without dementia [2]. These problems come with health, financial and social consequences for both the PLWD and their carers, including influencing residential care home admission [3]. Despite this, no interventions designed to minimise these problems for people living at home have been found to be effective [4] and it is acknowledged that these PLWD/carer dyads are poorly supported [5, 6]. A number of interventions have been evaluated but have failed to demonstrate an improvement in outcomes; these have predominantly focused on strategies to support toilet-use such as prompted voiding or scheduled toilet-use [4]. Lack of efficacy is perhaps not surprising as incontinence problems for PLWD and their carers are complex, comprising dementia, physical, psychosocial, societal and care system factors, with considerable contextual variation (e.g. relationship between PLWD and carer, type of dementia and degree of incontinence) [6].

By contrast, in other areas of dementia care complex interventions that consider individual dyad’s circumstances, needs and goals have been developed and shown promise (e.g. manualised, tailorable interventions to tackle sleep problems for PLWD living at home [7]). Evidence-based care to support PLWD/carer dyads to cope with incontinence appears to be lagging behind other areas (e.g. sleep management) thus providing the opportunity to learn from these more advanced domains. Some key findings from dementia research have concluded that

- interventions frequently fail to demonstrate effective understanding of the complexities of living with dementia [8],
- the amount of burden someone feels is individual, with different triggers and thresholds of burden for different carers [9],
- whether a program works may well depend on whether its focus matches carer needs [10],
- factors increasing the amount of burden experienced by carers include being female, the carer’s mental and physical condition, loss of family and peer support, and coping style [11] and
- the carer’s assessment of their role, how supported they feel and the level of functioning of the PLWD determined the perception of burden [12].

These findings need to be considered when developing interventions for managing incontinence.

Ideally, interventions would support PLWD to regain their continence. Many PLWD are unlikely to regain independent continence (dry, not dependent on on-going treatment) as their disease progresses, and for frail older people, dependent continence (dry with assistance) and contained incontinence (dry with products such as disposable absorbent pads) are seen as the treatment goals where independent continence is not achievable [13]. Moreover, it has been hypothesised that the poor containment of incontinence (rather than incontinence itself) is a key factor influencing carer strategies and their decisions regarding care home admission [14]. The primary analysis [6] of the data on which this paper is based addressed the causes, consequences of problems associated with dementia and incontinence and listed a number of potential solutions proposed by participants. In this paper, we present the results of a secondary analysis of the data investigating whether attaining well-contained incontinence diminishes the negative impact of living with incontinence for PLWD and their carers (and conversely whether poorly contained incontinence increases the negative impact).

## Objective

To investigate whether well-contained incontinence lessens the negative impact of living with incontinence for PLWD in the community and their carers.

## Methods

Design: This paper reports on the secondary analysis of a qualitative study to establish the causes, consequences and potential solutions of incontinence and dementia at home.

Data Collection: Semi-structured interviews were undertaken with PLWD, carers and nurses (continence or dementia, registered or non-registered nurses). PLWD and carers who had experienced (or cared for) incontinence problems were recruited via www.joindementiaresearch.nihr.ac.uk and via dementia/carer groups. Nurses were recruited via their employers (two NHS Community Trusts and Dementia UK). Data collection took place from January to October 2019. A purposive sampling approach was used to capture data from a range of participants (by sex, dyad relationship and living arrangements for PLWD/carer participants and professional background for HCP participants). Recruitment continued until no new major themes arose for two consecutive interviews for the primary analysis. Interviews were digitally recorded and transcribed verbatim. Unique participant codes were used, and any identifying details removed.

Data Analysis: For this secondary analysis, a framework approach was used to identify themes based on the level of containment and the degree of impact of incontinence on the lives of the PWLD and carer. The framework steps of familiarisation, constructing a thematic framework, indexing and sorting, data summary and display and mapping and interpretation were followed [15].

The Consolidated Criteria for Reporting Qualitative (COREQ) Research guideline statement assists the reporting [16]. Ethical Approval was received from NHS Health Research Authority, London—City & East Research Ethics Committee (reference 18 LO 1836). This research was supported by funding from Alzheimer’s Society (grant number AS-JF-17-012).

**Table 1:** Summary of participants

**Group**	**Sex**	**Details**	
**PLWD**		**Relationship with carer**	**Place of residence when care took place**
PLWD 1	F	Wife of current carer (H6)	With carer
PLWD 2	M	Husband of current carer (W10)	With carer
**Carer**		**Relationship with PLWD**	
H1	M	Husband to wife (current carer)	With PLWD
H2	M	Husband to wife (current carer)	With PLWD
H3	M	Husband to wife (current carer)	With PLWD
H4	M	Husband to wife (current carer)	With PLWD
H5	M	Husband to wife (former carer)	With PLWD
H6	M	Husband to wife (current carer)	With PLWD
H7	M	Husband to wife (current carer)	With PLWD
W1	F	Wife to husband (former carer)	With PLWD
W2	F	Wife to husband (former carer)	With carer
W3	F	Wife to husband (current carer)	With PLWD
W4	F	Wife to husband (current carer)	With PLWD
W5	F	Wife to husband (current carer)	With PLWD
W6	F	Wife to husband (former carer)	With PLWD
W7	F	Wife to husband (former carer)	With PLWD
W8	F	Wife to husband (former carer)	With PLWD
W9	F	Wife to husband (former carer)	With PLWD
W10	F	Wife to husband (current carer)	With PLWD
N1	F	Niece to aunt (former carer)	Separate from PLWD
S1	M	Son to father (former carer)	Separate from PLWD
S2	M	Son to mother (current carer)	With PLWD
Sil1	M	Son-in-law to mother-in-law (current carer)	Separate from PLWD
D1	F	Daughter to mother (current carer)	Separate from PLWD
D2	F	Daughter to mother (former carer)	With PLWD
D3	F	Daughter to mother (former carer)	With PLWD
D4	F	Daughter to father (former carer)	Separate and then with PLWD
D5	F	Daughter to mother (former carer)	Separate from PLWD
Dil1	F	Daughter-in-law to mother-in-law (former carer)	Separate from PLWD
**Nurse**		**Specialism, registered/non-registered**	
DemN1	F	Dementia, registered	
DemN2	F	Dementia, registered	
DemN3	F	Dementia, registered	
DemN4	F	Dementia, registered	
DemN5	F	Dementia, non-registered	
DemN6	F	Dementia, registered	
DemN7	F	Dementia, registered	
DemN8	F	Dementia, registered	
ConN1	F	Continence, non-registered	
ConN2	F	Continence, registered	
ConN3	F	Continence, non-registered	
ConN4	F	Continence, non-registered	
ConN5	F	Continence, non-registered	
ConN6	F	Continence, non-registered	
ConN7	F	Continence, registered	
ConN8	F	Continence, registered	
ConN9	F	Continence, registered	

## Results

Forty-five people (twenty-six family carers, two people with dementia, nine continence nurses and eight dementia nurses) took part. A summary of participants including place of carer residence and relationship with the PLWD is given in Table 1. Full details of the primary analysis on the causes and consequences of and potential solutions for dementia-associated continence problems are provided elsewhere [6]. Due to the low number of PLWD recruited to the study, the experiences of PLWD included here are largely second-hand, but many carers and HCPs reported the responses of PLWD either verbatim or with substantial knowledge of the person and their opinions.

All PLWD or carer participants described experiences of using disposable absorbent products to contain incontinence. Many combined these products with toilet-use (with or without assistance).

This paper neither discusses in detail the reasons for well-contained or poorly contained incontinence (factors such as type and degree of incontinence, appropriate product use, co-morbidities or stage of dementia) nor does it seek to quantify the impact of incontinence. Instead, this paper focuses on investigating experiences of the influence of well- or poorly contained incontinence on the lives of PLWD and their carers.

### Definition of key concepts

During the process of summarising the data, working definitions of key concepts were developed as follows:

- Well-contained incontinence: reported as infrequent (once or twice a week or less) usually minor leakage beyond pad (e.g. limited to underwear or small amount on personal clothes).
- Poorly contained incontinence: reported as frequent (daily or multiple times per week), often substantial leakage beyond pad (e.g. soiling clothes, furniture, carpets).
- Lower impact of incontinence: incontinence care perceived as manageable. It might still be time-consuming and limit activities but was not considered to be a major stressor to either member of the dyad.
- Higher impact of incontinence: managing incontinence was perceived to be a major negative influence, often one of the most or the most difficult part of the caring experience for one or both members.

### Categories of participants’ experiences

Using these definitions, participants’ experiences were categorised as outlined in the methods and examples of those experiences were placed into one of four themes:

- Group 1. Well-contained incontinence, lower negative impact on both members of the dyad.
- Group 2. Well-contained incontinence, higher negative impact on either or both dyad members.
- Group 3. Poorly contained incontinence, higher negative impact on either or both dyad members.
- Group 4. Poorly contained incontinence, lower negative impact on both members of the dyad.

Many participants described different incontinence trajectories where either the level of containment or degree of impact on the dyad changed along the weeks, months or years of care. In these cases, the experiences of one dyad could be assigned to more than one Group during different time periods.

### Main findings

**Figure 1:**
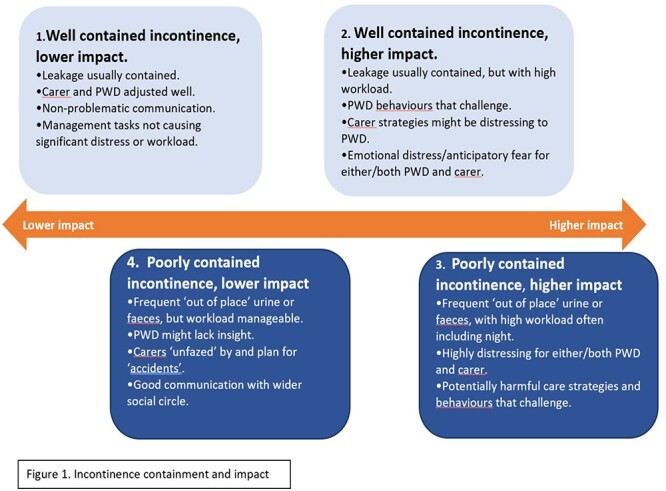
Incontinence containment and impact.

Together, these themes form the preliminary model illustrated in Figure 1. The model presents the four groups on a continuum from low to high negative impact caused by incontinence. The well-contained incontinence groups (1 and 2) are further towards the lower end of the spectrum than the poorly contained groups (3 and 4). However, Group 2 is worse affected than Group 4, and overall, poorly contained incontinence had a higher impact in terms of workload and distress than well-contained incontinence. The model does not aim to quantify the size of each group. Groups 1, 2 and 3 were more dominant, with fewer participant experiences meeting the criteria for Group 4. Common characteristics of each group are summarised below.

1. **Characteristics of Group 1 (Well-contained incontinence, lower negative impact)**

- In this Group, the challenges of incontinence care were considered manageable and practical with continence tasks a relatively straightforward part of the daily care routine. One husband commented: ‘I call her my big baby, it’s no different from when the children were young and I used to change their nappies.’ (H6). The process of care had little or no negative impact on the dyad’s relationship. Indeed, a small number of carers believed the experience of providing hands-on continence care enhanced their relationship, with one daughter remarking: ‘If anything we became closer. I absolutely adored her. It was a massive privilege to be able to help her with those needs.’ (D2). Some dyads used humour as a coping strategy: ‘She taps her bum as if to say got it on. So we get round it like that. We have a little game with it if you like.’ (H2).
- Many participants who felt they had adapted easily to managing incontinence mentioned the importance of their open communication style or relaxed relationship, commenting that they had an open relationship where bodily functions were easily discussed.
- Lack of negative response (for example absence of disgust, embarrassment or fear) appeared to help people to cope. This seemed to be a natural response, rather than a conscious strategy, even when dealing with faeces: ‘It didn’t ever make me squeamish, it was just how it was.’ (D2)

1. **Characteristics of Group 2 (Well-contained incontinence, higher impact)**

- Other carers, despite reporting generally well-contained incontinence, perceived a high negative impact associated with managing the symptom. Some PLWD had incontinence or toilet-use behaviours found challenging by their carers; equally some carers used strategies that PLWD appeared to find distressing. Many PLWD developed repetitive habits or other behaviours around toilet or product use, for example repeatedly going to the toilet to avoid ‘accidents’. Other PLWD declined or ‘resisted’ personal care associated with incontinence and this could become a battleground within their relationship: ‘The problem is she’s getting very resistant. So much so that it takes two people now. So I let the carers do it and I stand at the door of the bathroom so she can’t get out.’ (H1). HCPs recognised both the vulnerability of the PLWD and the dilemma faced by the carer to either compel the PLWD into receiving unwanted care or to leave hygiene needs unmet with the associated potential for harm.
- Even when continence was generally well-contained, emotional distress was frequently experienced by PLWD and carers. Carers often described PLWD’s experience of feeling humiliation: ‘For my wife it’s so humiliating because she hates it [receiving support with managing contained incontinence]. She won’t speak to people about it, it upsets her too much.’ (H3). Among carers who found incontinence care challenging, a common emotion was disgust (although this word was rarely used). There were two key categories: disgust with managing bodily waste (‘I mean I never changed my children’s nappies because I couldn’t cope with it, it just made me retch, but I’ve had to deal with it’ H5) or disgust with the change in the nature of the interpersonal relationship (‘I could do it for other people but I can’t do it for him [husband]. I have done it in emergencies but by choice, I don’t do it.’ W3). Additionally, if the PLWD had insight into their incontinence, the carers sometimes felt an additional burden, as they were dealing with the distress of the PLWD as well as their own.
- Incontinence care could trigger or exacerbate an unwanted change in the relationship, commonly there was a move to the carer adopting a parental or assertive position. This could be spouse caring for spouse or adult child caring for a parent as observed by a daughter: ‘I found it very strange because suddenly I was seeing my father naked. I’d never seen my father like that.’ (D4). The sex of the PLWD and the carer was often described as influencing the nature of care provided, particularly with mother and son dyads. One son who lived with his mother explained that he could prompt her and help her to reach the toilet, but he would not be able to help with hands on continence care and they would need residential care at that point.
- In contrast to Group 1, some dyads in Group 2 found communication barriers made incontinence care more challenging. A niece caring for her aunt said: ‘I never know whether she’s wearing them or not [continence pads]. I don’t like to pry. I suppose you pry more the closer the relationship is? I’m a niece and she’s my Aunt. They were brought up very strictly, very Victorian, so some of that rubbed off in my mum’s parenting of me.’ (N1).
- For some (both PLWD and carers), the fear of ‘accidents’ even though continence was generally well managed had a high impact. Despite only sporadic leakage, one husband said that his wife was anxious about risking possible humiliation: ‘We didn’t go out for meals, we didn’t go out to the pub, we didn’t go out to the cinema, we didn’t go to the theatre.’ (H5)
- Containing incontinence created a very high mental and physical workload for some carers, even without leakage from products. Carers spoke of the need for constant vigilance required to maintain containment. A daughter described her experience, ‘I seemed to spend my entire life in the toilet, up at 7am every morning straight into the bathroom and then at 9pm at night she would be put to bed, but then she’d start shouting to go and I’d be up probably every hour or two hours.’ (D3).

1. **Characteristics of Group 3 (Poorly contained incontinence, higher impact)**

- This Group experienced many of the challenges found in Group 2 (including emotional distress, anxiety, potentially harmful care strategies and changes in relationships), but further exacerbated by the challenges of poorly contained incontinence.
- For some, poor containment marked the end of many social activities or prevented the use of services, as one wife described: ‘He would spend days at a day centre, but he wouldn’t go to the toilet for them, so when I picked him up he had quite wet trousers, and then they did say they could no longer have him with the incontinence. So they had to finish and it had given me respite.’ (W1).
- If containment was not effective at night, this could have a substantial impact on the carer’s ability to cope: ‘I’ve had a few carers overnight which have said they’ve had to change their loved one two or three times because the pad hasn’t been able to absorb the full volumes of urine or the bed has been soaked.’(DemN1)
- Poor containment added considerably to the workload of carers in terms of cleaning floors, furniture and dealing with laundry: One daughter said, ‘Every day two loads of washing just her clothes and her bedding.’ (D2). It could also impact on the home environment, particularly regarding smell: A daughter who lived separately from her mother described her experience, ‘There was one Saturday morning quite recently where we’d been to her bathroom, and the smell [of urine] when we got into the flat was enough to knock you down’ (D1).
- In a small number of cases, carers admitted that the frustration they experienced with poor containment was expressed in ways that they regretted including shouting at the PLWD, ‘I’m not the best person in the world and I get frustrated. I don’t have the bedside manner on me. You will do as you are told because at the end of the day I’ve got to get her cleaned up and she doesn’t want to be cleaned up, I mean there was mess all over the carpet.’ (H4).
- For some, ‘out of place’ incontinence, particularly faecal incontinence, marked the end of their ability to care. One wife stated that she could not cope with the thought of clearing up her husband’s faeces: ‘Then I realised he was doing something else on the floor as well as wee and then I realised I couldn’t do it anymore. The wee I could cope with but not the other’ (W7). Incontinence is clearly seen by HCPs as an issue that impacts on both members of the dyad and their home environment and that it is often a step too far for carers: ‘It’s massive. I think it’s at that point that people give up because it’s just too much. Put the two together and you’ve got a demented person who is pooing all over the place. Who wants to deal with that?’ (DemN4). The occurrence of crisis events (such as widespread faeces) can cause significant distress for PLWD and carers, as a nurse recalled: ‘She spread it all around her flat, it was all in the bathroom, all in the lounge on the floor, she was on the floor in the bedroom in the foetal position and her daughter came in and found this flat that’s usually immaculate covered in faeces and thought, oh my God what the hell has happened.’ (DemN3).

1. **Characteristics of Group 4 (Poorly contained incontinence, lower impact)**

- Group 4 participants reported less impact than Group 3, but more than Group 1 (due to the requirement for cleaning and washing caused by leakage). PLWD in this Group often lacked insight into the problems associated with their poorly contained continence and some carers believed that this helped them to cope. Some carers in this group appeared to be less worried about what other people think or find generally supportive that helped them to continue with their normal social activities. When talking about observable incontinence outside the home, one wife said, ‘Well stuff them [other people]’ (W10). One man cared for his wife who had regular heavy episodes of incontinence and described an incident on holiday, ‘Completely unannounced she stood up and just wet herself. It was like a horse to be honest. But we go there so often and they’re so nice there it didn’t faze them at all. They said oh don’t worry about it and then they went and got some mops and buckets and cleaned it all up and even gave us a tablecloth to use in the taxi on the way home.’ (H7). The same husband knew that his wife might be in a supermarket with wet clothes, said, ‘I just take her out. No one even knows, you are walking around all the time and there are plenty of others in there I can assure you probably in a worse state.’ (H7). The response of services outside of the home was also important to maintaining activities. In contrast to the wife quoted in Group 3, this wife described the proactive approach of her husband’s day centre: ‘Oh gosh, they did have a big problem with him one day. It was with incontinence, but they always have a spare pair of trousers. I can’t think what happened but she said, don’t worry we’ll still be taking care of him.’ (W5).
- Some dyads accept the potential leakage but plan carefully to limit the consequences, particularly so they can continue to go out, as one wife described: ‘We always have three pads, I have gloves in case it’s a poo, we have the disposable bags and we have baby wet wipes all in a bag and he would put that in his backpack as we go out. If we’re going to someone’s house we always take a disposable [pad] and a towel and I put that on their chair so there’s no way that they are going to get a wet chair.’ (W5).
- As in Group 1, if the participant reported good levels of communication, even regarding difficult topics, it appeared to ease the burden of incontinence. One daughter-in-law explained that her mother-in-law, regularly had ‘accidents’, but the extended family all knew so they could still see them without fear: ‘We’ve always talked about everything and it’s like we always talk to our kids about everything so I think that’s just – but some people don’t talk about anything do they? That’s like a blackline you don’t go across.’ (Dil1).
- Some carers in this Group recognised that strategies to maintain containment sometimes caused more harm than good, for example leading to conflict, as one daughter who lived separately from her mother explained: ‘I reminded her [to change her pad] in my lunch hour and she’d get really cross with me and we’d end up having an argument and I just gave up, I stopped doing it because it was more distressing for her and irritating for me [than “accidents”].’ (D5).

Participants did transition between groups. For some, this was a positive move, for example from Group 3 to Group 4 once they had adapted to living with the incontinence, as illustrated by one wife’s comments: ‘It really upset me but it’s part of life now, isn’t it? We don’t even think about it.’ (W5). For others, containing incontinence became more challenging (e.g. faecal incontinence developed in addition to urinary or toilet-use behaviours became more challenging) and they moved from Group 1 or 2 down to Group 4, or more commonly Group 3, as described by another wife’s comment: ‘I’ve just told the family we really can’t come and stay anymore because I’ve no idea what’s going to happen at night now and I can’t have that happening in their house however good they are. They say, oh it doesn’t matter. I’m thinking, it does actually. But no we’ve stopped going away completely.’ *(W3).*

## Discussion

Reliable containment is an important goal for PLWD living at home and their carers, but it is not the only goal. Containment is not the only factor influencing the degree of negative impact on PLWD and carers. Well-contained incontinence can still be a considerable problem for one or both members of the dyad. Indeed, in some circumstances, the benefits of achieving well-contained incontinence are not worth the disadvantages of the strategies used. The factors found to influence the impact of incontinence (e.g. emotional impact, practical management, relationship changes, behaviours that challenge, presence of faecal incontinence) have mostly been observed previously [14, 17, 18]). Likewise, the variation in the burden of care perceived by carers of PLWD in ostensibly similar circumstances is reported in existing literature [9]. However, this study builds on existing work, providing improved understanding of why similar levels of continence containment (both well-contained and poorly contained) can lead to varying degrees of negative impact. This insight will support the design of interventions with potential to reduce the negative impact incontinence (whether well or poorly contained).

Existing literature has highlighted that carers of PLWD in similar circumstances can cope very differently [19] due to a number of factors including coping style and cognitive strategies used [20]. For example, carers who use emotional support and acceptance-based strategies (e.g. getting emotional support from others, positive reappraisal, trying to see the bright-side) experience less anxiety compared with those using dysfunctional strategies (e.g. wishful thinking, denial) [20]. Variation in coping styles and cognitive strategies can be seen between the Groups in this paper, for example willingness (or otherwise) to accept and plan for the risk of ‘out of place’ urine or faeces in public. However, in the case of incontinence management, there is an additional layer of negative emotions influencing ability to cope, including disgust and shame.

The emotional cost of managing incontinence and the negative impact it can have on lives have been previously observed [21, 22]. This study found substantial variation in the extent of the emotional impact on PLWD and carers. Disgust (repulsion with managing incontinence) was expressed by many carers, particularly in relation to faeces. Predominantly, this was associated with seeing, smelling or clearing up urine or faeces, particularly when ‘out of place’ for example with faecal smearing. Rozin *et al.*’s Model of Disgust (1987) describes this as Core Disgust (disgust with bodily products) [23]. However, the Interpersonal and Moral Disgust [23] was also expressed when urine or faeces were associated with the ‘wrong’ person. For example, several participants felt capable of providing incontinence care for some people but not others due to their relationship. This was most commonly expressed by sons regarding their mothers or daughters for their fathers, but also other relationships including spousal. For these participants, the urine and faeces were not the problem per se; it was the combination of urine/faeces and one particular person for whom they felt that they should not be providing intimate care. Poor disgust suppression has previously been associated with increased anxiety in the carers of people with neurodegenerative disease [24]. This might indicate that carers who experience low disgust suppression need additional help to cope with continence care.

Like disgust, shame has previously been associated with incontinence [25] and has also been linked to low levels of help-seeking [26]. In this study, carers reported that some PLWD felt shame at receiving incontinence care and were fearful of the consequences (e.g. being seen with ‘out of place’ urine or faeces or being placed in residential care). It also provided examples of carers experiencing vicarious shame. Welten *et al.* [27] describe two processes of how another’s behaviour links to self: group-based where an in-group member threatens a person’s social identity (e.g. the PLWD has an ‘accident’ in a public place and the carer feels shame by association) and empathy-based shame where the carer imagines himself or herself in the PLWD’s position. While there is a body of literature on incontinence-associated disgust, shame and stigma, stigma reduction interventions (available for other conditions such as mental ill-health or obesity) are currently lacking.

Variation in the degree of negative impact associated with incontinence (whether well- or poorly contained) also seemed to be linked with the absence or presence of behaviours of PLWD that challenged carers. These behaviours broadly fit into the two categories identified as challenging by family carers in a meta-ethnographic synthesis by Feast *et al.* [28]: (i) Changes in communication and relationship (e.g. aggression when dealing with continence care) and (ii) perceptions of transgressions against social norms (e.g. ritualistic behaviours around toilet-use). Consideration is required of how to relieve the causes of these behaviours and how to support carers to cope. Non-consensual care was also found to be an issue with a small number of dyads. Previous research highlights the need for carers to speak to HCPs to help find common ground and alternatives to involuntary treatment [29]. Ostaszkiewicz’s conceptual model of the risk of elder abuse posed by incontinence and care dependence [30] expands on the potential causes and outcomes of coercion, chastisement and neglect that have the potential to provide a foundation for interventions to address this issue.

The preliminary model proposed in this paper has helped to clarify that while improving and maintaining effective continence containment is a fundamental goal for interventions to support people living with dementia and incontinence, and there are a range of other issues that must also be addressed. When developing new interventions, this model could support the application of the findings from previous dementia intervention research outlined in the Introduction. Any new intervention should aim to support and protect both the PLWD and their carer within the context of highly variable circumstances, personalities and relationships.

This study is not without limitations. The key weakness is the lack of PLWD participants. Although carers and nurses provided detail (often verbatim) on conversations with people they cared for, this cannot replace the direct perspectives of PLWD. Secondly, the sample was entirely white British and does not capture a full diversity of views, and this might be of particular importance when considering cultural variations, including in shame and disgust. Finally, the categorisation of participants’ experiences into four groups was subjective and not participant checked. The concept of what is well or poorly contained incontinence warrants further exploration with PLWD and their carers. Despite these limitations, this method has allowed the development of a preliminary model, ready for further development and evaluation.

## Conclusion

Reliable containment is not the only goal for people living with the effects of both dementia and incontinence. Other factors including coping strategies, negative emotions, behaviours that challenge and coercive care strategies can influence the extent of the negative impact of incontinence, whether well- or poorly contained. This paper presents a preliminary model describing the relationship between continence containment and the negative impact incontinence has on PLWD and their carers ready for further development and evaluation. The model indicates that the objective of future interventions should be both to improve the likelihood of achieving containment and to reduce the impact of managing incontinence on the day-to-day lives of dyad members.
